# Transplacental Zika virus transmission in *ex vivo* perfused human placentas

**DOI:** 10.1371/journal.pntd.0010359

**Published:** 2022-04-20

**Authors:** Thomas Langerak, Michelle Broekhuizen, Peter-Paul Alexander Unger, Lunbo Tan, Marion Koopmans, Eric van Gorp, A. H. Jan Danser, Barry Rockx

**Affiliations:** 1 Department of Viroscience, Erasmus Medical Center, Rotterdam, the Netherlands; 2 Department of Internal Medicine, Division of Pharmacology, Erasmus Medical Center, Rotterdam, the Netherlands; 3 Department of Pediatrics, Division of Neonatology, Erasmus Medical Center, Rotterdam, the Netherlands; NIAID Integrated Research Facility, UNITED STATES

## Abstract

A Zika virus (ZIKV) infection during pregnancy can result in severe birth defects such as microcephaly. To date, it is incompletely understood how ZIKV can cross the human placenta. Furthermore, results from studies in pregnant mice and non-human primates are conflicting regarding the role of cross-reactive dengue virus (DENV) antibodies on transplacental ZIKV transmission. Elucidating how ZIKV can cross the placenta and which risk factors contribute to this is important for risk assessment and for potential intervention strategies for transplacental ZIKV transmission. In this study we use an *ex vivo* human placental perfusion model to study transplacental ZIKV transmission and the effect that cross-reactive DENV antibodies have on this transmission. By using this model, we demonstrate that DENV antibodies significantly increase ZIKV uptake in perfused human placentas and that this increased uptake is neonatal Fc-receptor-dependent. Furthermore, we show that cross-reactive DENV antibodies enhance ZIKV infection in term human placental explants and in primary fetal macrophages but not in primary trophoblasts. Our data supports the hypothesis that presence of cross-reactive DENV antibodies could be an important risk factor for transplacental ZIKV transmission. Furthermore, we demonstrate that the *ex vivo* placental perfusion model is a relevant and animal friendly model to study transplacental pathogen transmission.

## Author summary

Zika virus is a mosquito-transmitted virus that can cause severe birth defects such as microcephaly when the infection occurs during pregnancy. Understanding how Zika virus crosses the placenta during pregnancy is important for future prevention strategies for vertical Zika virus transmission. Despite significant efforts to study this, to date it remains incompletely understood how Zika virus can cross the placenta and which risk factors contribute to this form of transmission. In this study we use an *ex vivo* placental perfusion model to study transplacental Zika virus transmission. The *ex vivo* placental perfusion model is a highly physiological and animal friendly model that mimics the *in vivo* conditions during pregnancy. We found that antibodies against the closely related dengue virus can significantly enhance placental uptake of Zika virus and Zika virus infection of human placental explants and fetal macrophages. These findings indicate that presence of cross-reactive dengue virus antibodies could contribute to transplacental Zika virus transmission.

## Introduction

Zika virus (ZIKV) is a mosquito-transmitted virus from the *Flaviviridae* family, genus flavivirus. A ZIKV infection during pregnancy can result in severe birth defects such as microcephaly and in fetal loss [1,2]. It is estimated that this occurs in 2–20% of ZIKV infections during pregnancy [3,4]. To date it remains incompletely understood how ZIKV can cross the placenta which normally serves as an important physical and immunological barrier to prevent maternal-fetal pathogen transmission [5]. More insight on how ZIKV crosses the placenta and risk factor identification is important for risk inventory and to create prevention strategies for transplacental ZIKV transmission.

The human placenta consists of many chorionic villi, which are tree-like structures that are the functional units of the placenta. These villi are either floating in maternal blood in the intervillous space or are anchored to the maternal part of the placenta (decidua) through extravillous trophoblasts. Multinucleated syncytiotrophoblasts line the chorionic villi, forming a cellular barrier against pathogens. Cytotrophoblasts are located below the syncytiotrophoblasts and can differentiate in extravillous trophoblasts and syncytiotrophoblasts. The stroma of chorionic villi consists of fibroblasts, fetal macrophages (Hofbauer cells) and endothelial cells. From the second pregnancy trimester, maternal blood flows in the intervillous space where it comes into contact with the chorionic villi [6]. From this pregnancy stage, maternal immunoglobulin G (IgG) is actively transported across the placenta through neonatal Fc-receptor (FcRn) mediated transcytosis in syncytiotrophoblasts [7,8].

Presence of antibodies against dengue virus (DENV), a flavivirus that is closely related to ZIKV, has been suggested to be a risk factor for transplacental ZIKV transmission through FcRn-mediated transcytosis of ZIKV–DENV antibody immune complexes across syncytiotrophoblasts in the placenta. This mechanism of transplacental transport has previously been observed for cytomegalovirus (CMV) while for ZIKV, FcRn-mediated transplacental transcytosis has been observed in pregnant mice but not in pregnant non-human primates (NHP) [9–13]. A possible explanation for these conflicting results is that there are important structural differences between the rodent and NHP placenta such as the presence of trophoblasts giant cells in the rodent placenta while these cells are not present in the NHP and human placenta. Furthermore, deep trophoblast invasion, which in humans is essential for optimal nutrition of the fetus, is limited in especially the rodent- but also the NHP placenta [14,15]. Therefore, there is a need to study the possible harmful effects that cross-reactive DENV antibodies may have on transplacental ZIKV transmission in a model that better represents the human *in vivo* conditions during pregnancy.

In this study, we use the human *ex vivo* dual placental perfusion model to study the effects of cross-reactive DENV antibodies on transplacental ZIKV transmission. This physiologically representative and dynamic model is well suited to study placental uptake and transplacental transport with conserved structural integrity [16,17]. By using this model and human placental explants, we demonstrate that cross-reactive DENV antibodies enhance placental ZIKV uptake and infection.

## Methods

### Ethics statement

Placentas of women with uncomplicated singleton pregnancies who underwent an elective cesarean section were collected immediately after delivery at the Erasmus Medical Center, Rotterdam, the Netherlands. Patients with retained placenta, viral infections (HIV, hepatitis B, ZIKV), the presence of fetal congenital abnormalities and any form of diabetes were excluded. The study was exempted from approval by the local institutional Medical Ethics Committee according to the Dutch Medical Research with Human Subjects Law (MEC-2016-418 and MEC-2017-418). Written informed consent was obtained from all patients prior to donating their placenta for this study.

### Virus strain

The Asian lineage of Zika virus (Suriname 2016, GenBank KU937936, third passage) was used for all infection experiments. For the placental perfusion experiments, this virus was inactivated by incubation with 0.02% β-propiolactone (BPL, Ferak Berlin GmbH) for three days at 4°C followed by three hours incubation at 37°C to hydrolyze BPL. Inactivation of the virus was confirmed by inoculation of Vero cells with high concentrations of the inactivated virus for five days at 37°C which resulted in no infection based on absence of cytopathogenic effect (CPE) and staining of ZIKV E-protein assessed with fluorescent microscopy.

### Placental perfusion experiments

The *ex vivo* dual placental perfusion model used in this study was performed as described in detail by Hitzerd et al. and was located in a biosafety level 1 laboratory [18]. In short, placentas were collected directly after cesarean section and were attached to the perfusion machine. The double set-up of this system allowed for perfusion of two cotyledons at the same time if placentas had two cotyledons that were in sufficient condition to be attached to the perfusion machine. The maternal and fetal compartments were perfused with 200 mL Krebs-Henseleit buffer supplemented with 5000 IU heparin and 95% O_2_, 5% CO_2_ at 37°C. After an equilibration and washout period of approximately 60 minutes, ZIKV^BPL^ that was pre-incubated for 60 minutes with 333 μl pooled flavivirus naïve serum or pooled serum containing DENV-2 nAbs (both 1:250 dilution) was added to the maternal circulation in a concentration of 1x10^5^ TCID_50_ equivalent/mL. To block the interaction between immune complexes and FcRn, protein G was added to ZIKV^BPL^–immune complexes at a concentration of 3 μg/ml and 9 μg/ml, 60 minutes prior to adding the complexes to the maternal circulation. Placentas were perfused for either 40 or 120 minutes and samples from the maternal and fetal circulation were collected every 10 or 15 minutes, respectively, for ZIKV RNA detection with RT-PCR. After 40 or 120 minutes of perfusion, placentas were flushed with fresh perfusion medium in an open circulation for 10 or 30 minutes, respectively, to wash away residual, unbound ZIKV. Next, 4mm tissue biopsies were taken from the perfused cotyledon of the placenta after which the tissue was placed in a 4% formaldehyde solution for fixation for subsequent immunohistochemistry.

As a quality control for capillary leakage, which results in leakage of perfusion fluid from the fetal compartment to the maternal compartment, FITC-dextran (40kDa, 36mg/L) was added to the fetal circulation before start of perfusion. Samples were taken every 30 minutes from the fetal and maternal circulation and FITC-dextran levels in these samples were determined using a Multiwell Plate Reader (Victor X4 Perkin Elmer). In all perfusion experiments that are used for analysis, the fetal-to-maternal FITC-dextran ratio was below the cut-off for capillary leakage (0.03). Experiments in which capillary leakage did occur during perfusion were excluded from analysis. Antipyrine (100 mg/L) was added to the maternal circulation and detected in the fetal circulation using ultraviolet-visible spectroscopy (Shimadzu UV-1800) to confirm adequate overlap between the maternal and fetal circulation. ZIKV RT-PCR in the fetal circulation was only performed if the fetal-to-maternal antipyrine ratio was >0.65 after 120 minutes of perfusion, which was the case in 65% of the perfusions.

### ZIKV quantification

The amount of ZIKV in supernatants, expressed as 50% tissue culture infective dose per milliliter supernatant (TCID_50_/mL), was determined by 10-fold dilution endpoint titration on Vero cells and the method of Kärber was used to calculate TCID_50_ titers from three replicates [19].

For determination of ZIKV RNA in placenta tissue, the tissues were homogenized with a ceramic bead in a FastPrep-24 5G sample disruptor instrument (MP Biomedicals). ZIKV RT-PCR was performed with the 1086/1162c/1107-FAM primers/probes set described by Lanciotti et. al [20]. TCID_50_ equivalent values were extrapolated from a standard curve that was generated by making a 10-fold dilution of a ZIKV virus stock with a known virus titer ranging from 10^6^ TCID_50_ to 10^1^ TCID_50_.

### Human sera

Sera that were obtained from a previously performed ZIKV seroprevalence study in Suriname that did not contain ZIKV nAbs were tested for ADE potential with an *in vitro* ZIKV ADE assay using U937 cells (Department of Immunology, Erasmus MC, the Netherlands) as described previously [21,22]. Infection experiments with placental explants were performed with one serum containing DENV-2 nAbs which reached a peak ADE titer at 1:200 dilution. ADE was defined as a statistically significant increase in viral titer in supernatants in presence of serum containing cross reactive antibodies compared to conditions without serum or with control serum. Because of the relatively large volume of the perfusion medium (200 mL), placental perfusion experiments were performed with pooled serum from six donors. This pooled serum reached a peak ADE titer at 1:200 dilution. DENV-2 nAb titers were determined in these sera as described before and were >1:100 for all sera, see S2 Table [23]. Two sera in which no antibodies against all clinically relevant flaviviruses (among others ZIKV, DENV, yellow-fever virus, West Nile virus, tick-borne encephalitis virus and Japanese encephalitis virus) were detected with a protein microarray, were used as a negative control [24].

### Infection of human placental explants

Placentas were collected within 30 minutes after an elective caesarian section. Tissues were extensively washed with PBS to remove blood and antibodies. After the removal of the decidua and chorionic plate, tissues containing chorionic villi were cut into blocks of approximately 3x3 mm which were placed in a 24-wells plate in 1 mL of DMEM/F12 medium (Lonza) supplemented with 10% fetal bovine serum (FBS, Sigma), 5.7mL 7.5% Sodium Bicarbonate (Lonza) and 50 mg/mL Primocin (InvivoGen). Subsequently, the villi were infected with either 1.0x10^5^ TCID_50_/mL ZIKV, ZIKV+DENV nAb containing serum, ZIKV+1 μg/mL humanized 4G2 (hu4G2, IgG1, The Native Antigen Company) or ZIKV+flavivirus naïve serum in 750μl medium. At six days post infection (dpi), half of the tissues were homogenized for RNA isolation and ZIKV RT-PCR, and the other tissues were fixed in a 4% formaldehyde solution for immunohistochemistry and *in situ* hybridization (ISH).

For Fc-gamma receptor (FcγR) blocking experiments, explants were pre-incubated for two hours with monoclonal antibodies against FcγRs (clones 3G8, 6C4 and 10.1), 3 μg/well. After two hours, ZIKV^BPL^–DENV nAbs immune complexes were added to these tissues and incubated for another two hours after which the tissues were washed three times, and the explants were incubated in 1 mL culture medium for 48 hours. For blocking the interaction of IgG with FcRn, recombinant protein G (ThermoFisher, 1.5 μg/mL) was added to ZIKV^BPL^–DENV nAbs immune complexes and incubated for one hour at 37°C before adding this to placental explants for two hours. After two hours, explants were washed three times and incubated in 1 mL culture medium for 48 hours.

### ZIKV RNA detection with *in situ* hybridization and immunohistochemistry co-staining

ISH for ZIKV RNA was performed with RNAscope (Advanced Cell Diagnostics) on 5 μm thick slides of formalin fixed, paraffin embedded tissues, according to the manufacturer’s instructions and as described before [25]. Controls included a positive and negative control probe (ubiquitin C and DapB respectively) and uninfected placenta tissue on which the ZIKV probe was used. For immunohistochemistry, tissues were deparaffinized and rehydrated and boiled for 15 minutes in an EDTA buffer. Tissues were subsequently incubated with anti-cytokeratin-7 antibody (Abcam, ab52870, 1:350 dilution) followed by a HRP labeled goat anti mouse antibody (DAKO 1:100 dilution) to visualize trophoblasts. To visualize Hofbauer cells (HBCs), tissues were incubated with an anti-CD163 antibody (ThermoScientific, 1:100 dilution) followed with a rabbit anti mouse IgG antibody labeled with biotin (DAKO, E03354 1:100 dilution) followed by adding streptavidin-HRP (DAKO, D0397, 1:300 dilution). AEC (3-Amino-9-ethylcarbazole, Abcam) was used as HRP substrate, and counterstaining with Mayer’s hematoxylin (Merck) was performed for all tissues.

### Isolation of Hofbauer cells and trophoblasts

HBCs and trophoblasts were isolated as previously described before with few alterations [26]. After the decidua basalis was thoroughly removed and tissues were extensively washed with PBS, tissues were minced and incubated in digestion medium (RMPI-1640 medium (Lonza) supplemented with 10% FBS and 1 mg/mL collagenase IV (Worthington Biochem. Corp), 300 μg/mL DNAse 1 (Worthington Biochem. Corp)) at 37°C. Four digestion cycles of 30 minutes were performed and after each cycle, tissue was mechanically dissociated using gentleMACS Tissue Dissociator (Miltenyi Biotec). The cell suspension after the first digestion cycle, containing mainly dead cells and erythrocytes, was discarded. Mononuclear cells were isolated by using 30–70% Percoll density gradient centrifugation (GE Healthcare Life Sciences). HBCs were isolated from the interphase fraction using CD14-positive magnetic isolation as per manufacturer’s instructions (Miltenyi Biotec). The CD14-negative fraction containing cytotrophoblasts was collected and plated in a 96-wells plate that was treated with fibronectin and incubated overnight, after which non-adherent cells were washed away with fresh medium. Purity of the isolated cells, determined with confocal laser scanning microscopy, was >95% for HBCs and >80% for trophoblasts.

### Infection and visualization of Hofbauer cells and cytotrophoblasts

HBCs and trophoblasts were seeded at a density of 1.0x10^5^ cells per well and infected with ZIKV or ZIKV+DENV nAbs at a multiplicity of infection (MOI) of 0.5 for 48 hours. FcγR-blocking antibodies were added to the cells one hour prior to infection at a concentration of 10 μg/mL. In some conditions, protein G (1.5 μg/mL) was added to ZIKV–DENV nAbs immune complexes and incubated for one hour before adding this to the cells. For visualization with confocal laser scanning microscopy (Zeiss), cells were fixed and permeabilized (BD Cytofix/Cytoperm) and stained with primary antibodies against CD68 (DAKO, 1:75 dilution), cytokeratin-7 (Abcam, ab52870, 1:350 dilution) and 4G2 (Merck Millipore, 1:200) and secondary antibodies goat-anti mouse IgG1 A647, goat-anti mouse IgG2A AF488 and donkey anti-rabbit AF555 (all Invitrogen, 1:400 dilution). Nuclei were stained with Hoechst 33342 (Invitrogen). Percentage of infected cells and purity of cells were calculated using ImageJ. Processing of the confocal laser scanning microscopy images was done with the ImageJ plugin QuickFigures [27].

### Cytokine detection in supernatants

Cytokines were quantified in supernatants of primary placental cells with a 13-plex bead-based assay (Biolegend, LEGENDplex) according to the manufacturer’s instructions. Readout was performed with flow cytometry (BD FACSlyric), and data was analyzed with LEGENDplex data analysis software (Biolegend). Cytokine levels in supernatants of uninfected placental cells were compared to cytokine concentrations in supernatants of cells infected with ZIKV+control and ZIKV+DENV nAbs.

### Statistical analysis

Statistical analyses were performed using GraphPad Prism version 9.0 (Graphpad Software Inc). For non-normally distributed variables, the Kruskall-Wallis test with Dunn’s post hoc test was used while for normally distributed variables a one-way ANOVA with Dunnett’s post hoc test was used. For the placental perfusion experiments, ZIKV TCID_50_ equivalents/mL in the maternal circulation were compared at all time points using multiple t-tests with the Holm-Šidák correction for multiple testing. A P-value <0.05 was considered statistically significant.

## Results

### *Ex vivo* perfused placentas efficiently take up ZIKV immune complexes

**Fig 1:**
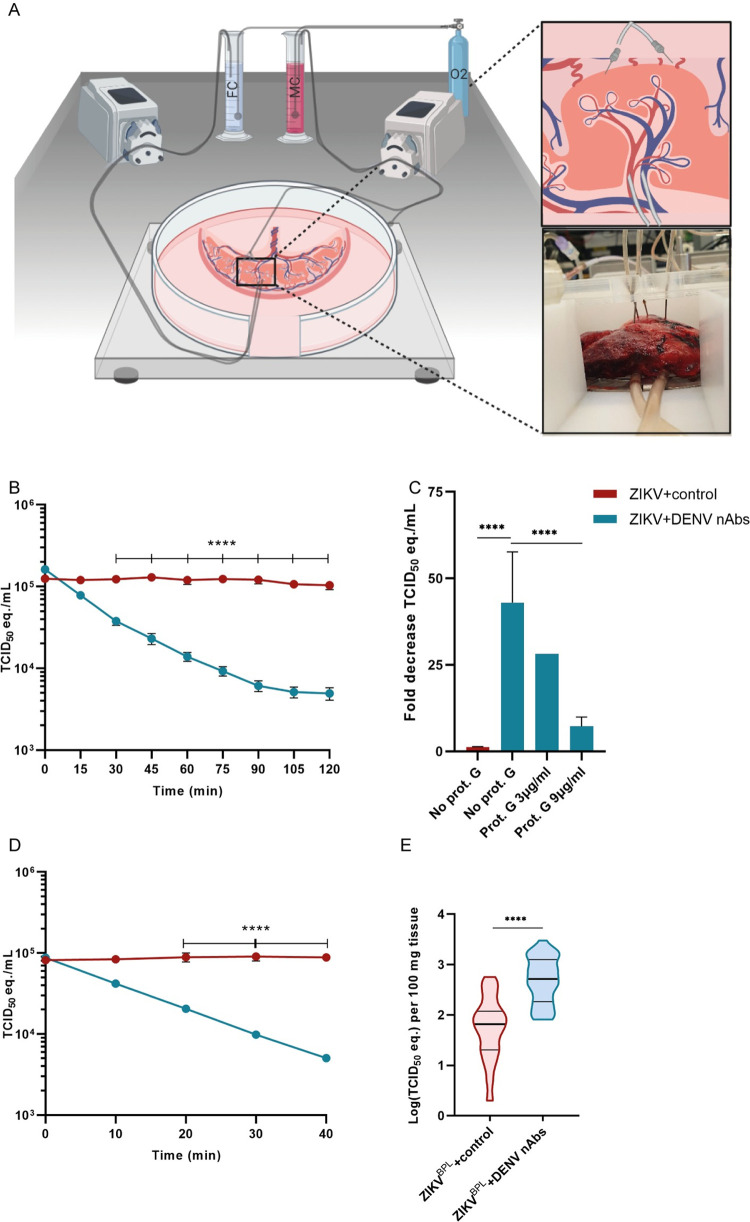
Efficient uptake of ZIKV immune complexes by *ex-vivo* perfused placentas. 1*10^5^ TCID_50_ equivalent/mL inactivated ZIKV (ZIKV^BPL^) was incubated with either flavivirus naïve serum or serum containing DENV-2 nAbs and added to the maternal circulation (MC) of the placental perfusion model and perfused for 40 or 120 minutes. **A:** Schematic overview of the *ex vivo* dual placental perfusion model. FC; fetal circulation. Created with Biorender.com. **B:** ZIKV RNA levels in the MC were determined every 15 minutes with RT-PCR up to 120 minutes to detect placental uptake of ZIKV^BPL^. Dots represent mean ±SEM, N = 3–4 donors per condition. **C:** Mean fold reduction (+SEM) in ZIKV TCID_50_ equivalent in the MC after 120 minutes of perfusion with and without different concentrations of protein G (Prot. G) to block the interaction of IgG with FcRn. N = 1–3 donors per condition. **D:** ZIKV RNA levels in the MC were determined every 10 minutes with RT-PCR up to 40 minutes. Dots represent mean±SEM, N = 2 donors per condition. **E:** ZIKV RNA was detected in tissue biopsies of the perfused placentas after 40 minutes of perfusion. Horizontal lines represent median and the 10^th^ and 90^th^ percentile cut-off. N = 2 donors per condition and 40 biopsies per condition. Data from the MC were analyzed with multiple t-tests with the Holm-Šidák correction and data from tissue lysates were analyzed with the Mann-Whitney U test. ****P<0.0001.

To study the role of DENV antibodies on placental uptake of ZIKV in a physiologically relevant model, we used the *ex vivo* dual perfused human placenta model. In this model, a cotyledon of the human placenta is attached to a perfusion machine directly after birth to recreate separate maternal and fetal circulations (Fig 1A). In comparison to placental explants, a commonly used model to study placental infection, the placental perfusion model has intact structural integrity and resembles the dynamic *in vivo* route of viral infection at the maternal-fetal interface better [16,28–30].

Placental perfusion experiments were performed with β-propriolactone (BPL) inactivated Asian lineage ZIKV (ZIKV^BPL^) because of biosafety regulations. We confirmed that ZIKV^BPL^ was replication incompetent but that the viral RNA could still be detected with RT-PCR and with ISH (S1 Fig). Experiments were performed with pooled serum containing DENV-2, but not ZIKV, neutralizing antibodies (ZIKV^BPL^+DENV nAbs) that was shown to optimally enhance ZIKV infection in U937 cells at a 1:200 dilution (S2 Fig). As a negative control, pooled serum without flavivirus antibodies was used (ZIKV^BPL^+control). Clinical data of participants from whom the placenta was used for perfusion are summarized in S1 Table.

A rapid reduction of ZIKV RNA levels was observed in the maternal circulation of placentas perfused with ZIKV^BPL^+DENV nAbs, indicating efficient placental uptake of ZIKV^BPL^ immune complexes (43.0-fold reduction in TCID_50_ equivalent/mL after 120 minutes, Fig 1B). ZIKV RNA levels remained stable in the maternal circulation of placentas perfused with ZIKV^BPL^+control (1.2-fold reduction in TCID_50_ equivalent/mL after 120 minutes, Fig 1B) indicating limited placental uptake of ZIKV^BPL^ in absence of DENV nAbs. In both conditions, ZIKV RNA could not be detected in the fetal circulation after 120 minutes of perfusion. To assess whether FcRn-mediated transcytosis of ZIKV immune complexes was the main mechanism of the efficient placental uptake of ZIKV immune complexes, protein G was added to the ZIKV immune complexes prior to placental perfusion. Protein G binds IgG at the FcRn binding domain (the hinge proximal region of the CH2 domain) and therefore blocks the interaction between FcRn and IgG but not between IgG and FcγRs [31,32]. Protein G reduced the placental uptake of ZIKV^BPL^+DENV nAb immune complexes in a dose dependent manner with a 7.2-fold decrease in ZIKV RNA levels in the maternal circulation with the highest protein G concentration compared to a 43.0-fold decrease without protein G after 120 minutes of perfusion (P < .001, Figs 1C and S3A).

After 120 minutes of perfusion and a 30-minute washout period with fresh perfusion medium to remove any residual unbound ZIKV, tissue biopsies were taken from the perfused cotyledon of the placenta. ZIKV RT-PCR was performed on the homogenized tissue biopsies and surprisingly, no difference was found in ZIKV RNA levels in placentas perfused for 120 minutes with ZIKV^BPL^+control, ZIKV^BPL^+DENV nAbs or ZIKV^BPL^+DENV nAbs+protein G (mean 1193 vs. 740 vs. 441 TCID_50_ equivalent/100 mg tissue, respectively, P>0.05, S3B Fig). We ruled-out that ZIKV^BPL^–DENV nAbs immune complexes adhered to the tubes of the placental perfusion machine by demonstrating that ZIKV RNA levels remained stable during circulation of ZIKV^BPL^+control and ZIKV^BPL^+DENV nAbs through the perfusion machine to which no placenta was attached (S3C Fig).

It has previously been demonstrated that BPL can successfully inactivate ZIKV while containing the antigenicity of the particle [33]. However, reduced viral membrane fusion has been observed with BPL inactivated influenza virus [34,35]. We hypothesized that if reduced viral membrane fusion would also occur after BPL inactivation of ZIKV, this can result in rapid breakdown of ZIKV^BPL^–DENV nAbs immune complexes in placental cells which could explain the lack of difference in ZIKV RNA levels in placentas perfused with ZIKV^BPL^+control compared to ZIKV^BPL^+DENV nAbs. Therefore, we performed shorter perfusions of 40 minutes followed by a washout period of ten minutes. Again, we observed rapid placental uptake of ZIKV^BPL^+DENV nAbs from the maternal circulation compared to ZIKV^BPL^+control (18.3 vs. 0.87-fold decrease in TCID_50_ equivalent/mL, respectively, P < .0001, Fig 1D). In these settings, we did detect significantly more ZIKV RNA in placentas perfused with ZIKV^BPL^+DENV nAbs compared to ZIKV^BPL^+control (average 773 vs. 117 TCID_50_ equivalent/100 mg tissue, respectively, P < .0001, Fig 1E). Collectively, these data suggest that placental uptake of ZIKV immune complexes is highly efficient and FcRn dependent. One possible explanation for the observation that after 40 minutes but not after 120 minutes of perfusion, more ZIKV RNA was found in placentas perfused with ZIKV^BPL^+DENV nAbs compared to ZIKV^BPL^+control is that BPL inactivation of ZIKV reduces the ability of endosomal escape resulting in lysosomal breakdown. We did, however, not investigate this hypothesis in detail in the current study.

**Fig 2:**
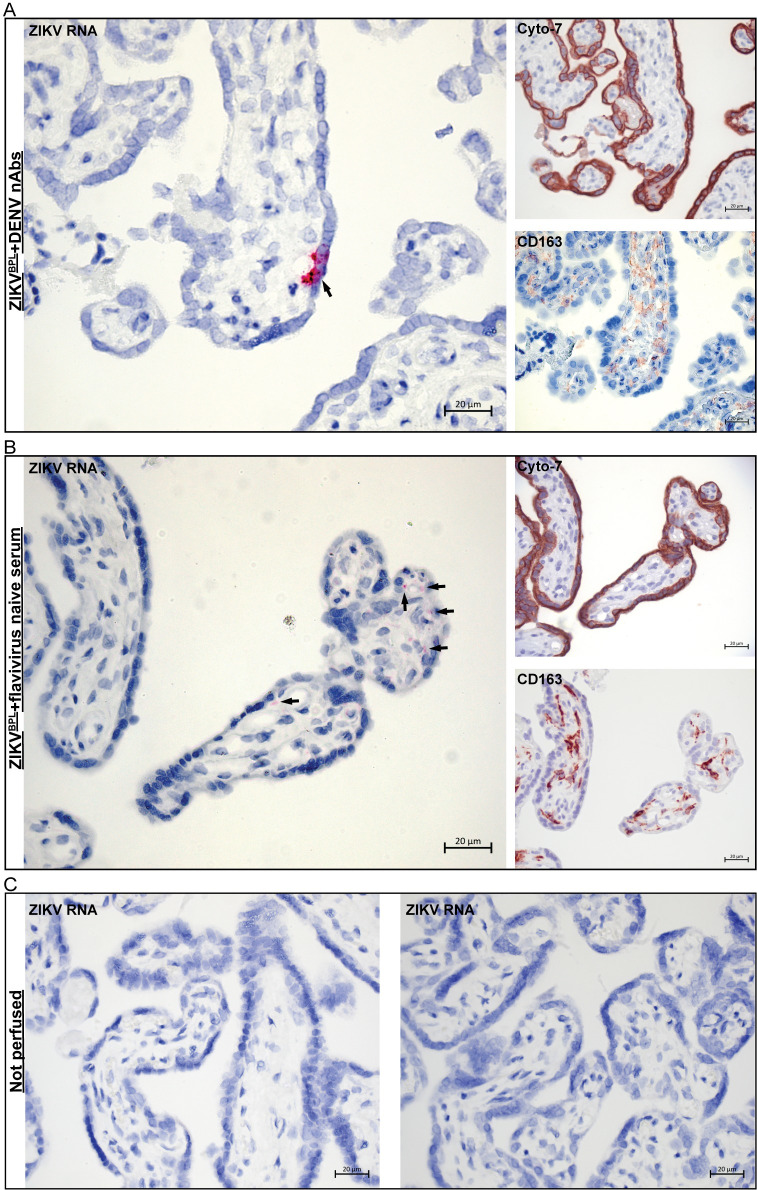
Detection of ZIKV RNA in chorionic villi of placentas perfused with ZIKV. ZIKV RNA was detected with ISH in formalin fixed, paraffin embedded tissue of perfused placentas. Chromogenic staining for cytokeratin-7 (cyto-7) and CD163 was performed in sequential slides to detect trophoblasts and Hofbauer cells, respectively. **A:** Staining for ZIKV RNA, trophoblasts and Hofbauer cells in a placenta that was perfused for 40 minutes with ZIKV^BPL^+DENV nAbs. **B:** Staining for ZIKV RNA, trophoblasts and Hofbauer cells in a placenta that was perfused for 120 minutes with ZIKV^BPL^+flavivirus naïve serum. **C:** Staining for ZIKV RNA in a placenta that was obtained directly after birth and was not perfused. Arrows indicate positive signal for ZIKV RNA.

Lastly, we performed ISH for ZIKV RNA on the perfused cotyledons to try to detect ZIKV RNA. As expected from replication incompetent virus, the signal intensity for ZIKV RNA was low. We did, however, sporadically detect ZIKV RNA in trophoblasts and chorionic villi in placentas that were perfused for 40 minutes with ZIKV^BPL^ +DENV nAbs and placentas perfused for 120 minutes with ZIKV^BPL^+control (Fig 2A and 2B) but not in non-perfused placentas (Fig 2C). These results suggest that ZIKV can cross the placental barrier of term placentas during *ex vivo* perfusion.

### FcRn-mediated ADE of ZIKV infection in placental explants

To confirm the results from the placental perfusion model with infectious virus, placental villus explants were isolated from term placentas and infected with ZIKV alone, ZIKV+DENV nAbs or ZIKV+control. As a positive control for ADE, ZIKV was pre-incubated with a humanized, monoclonal pan-flavivirus antibody (ZIKV+hu4G2) that is known to induce ADE of flavivirus infection *in vitro* [36].

**Fig 3:**
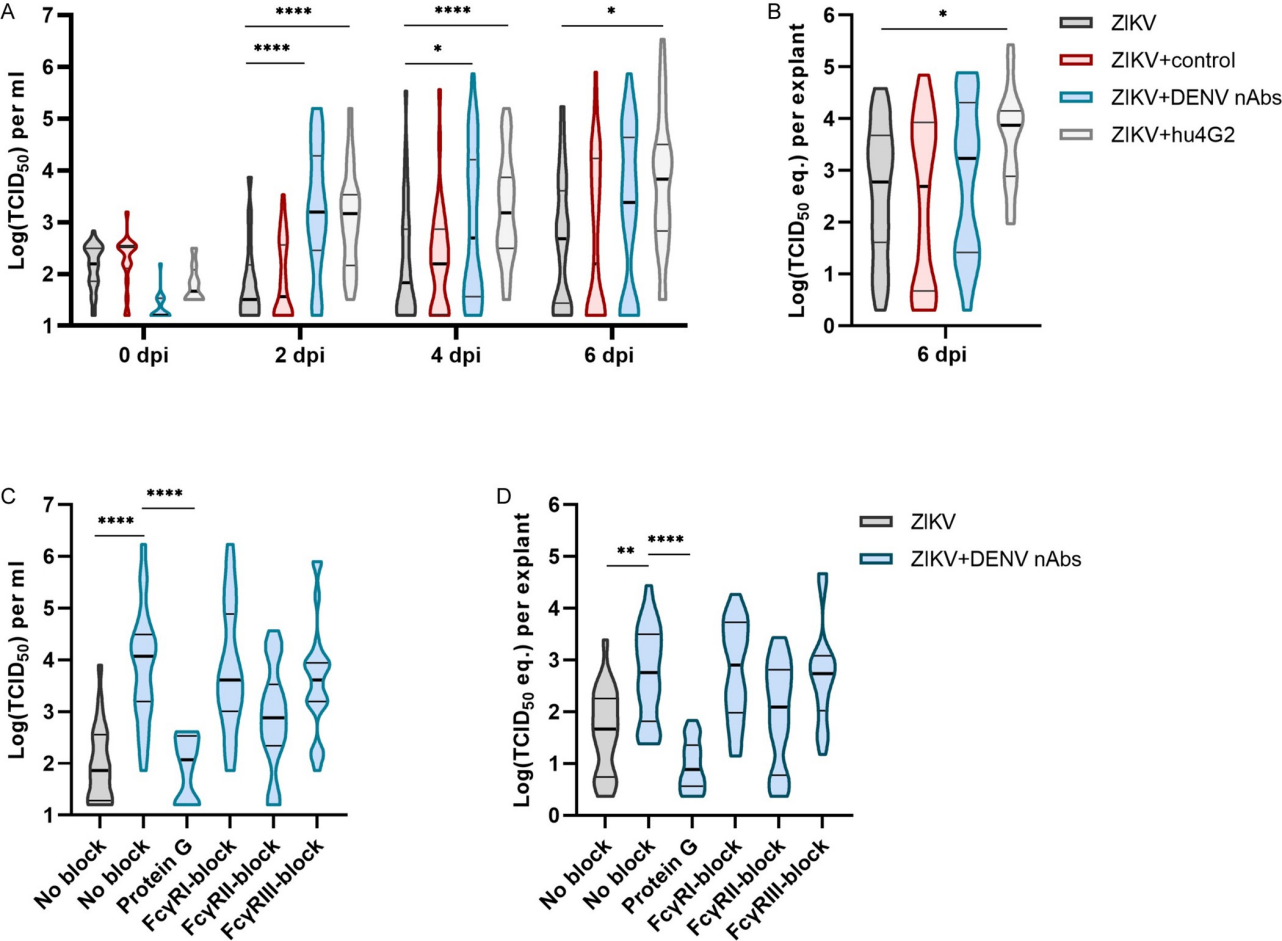
Cross-reactive flavivirus antibodies enhance ZIKV infection in placental explants. **A&B**: Term human placental explants were infected with 1.0x10^5^ TCID_50_/mL ZIKV alone or ZIKV that was preincubated with flavivirus naïve serum (ZIKV+control) or serum containing DENV-2 neutralizing antibodies (ZIKV+DENV nAbs), both in a dilution of 1:250, or with a humanized panflavirus monoclonal antibody (hu4G2, 1μg/mL). ZIKV titers were determined in supernatants **(A)** and ZIKV RNA levels were determined with RT-PCR in tissue lysates **(B)**. **C&D**: Term human placental explants were pre-treated with FcyR blocking antibodies or protein G was added to ZIKV–DENV nAbs immune complexes. Subsequently, the explants were infected with either 1.0x10^5^ TCID_50_/mL ZIKV or ZIKV+DENV nAbs. ZIKV titers were determined in supernatants at two dpi **(C)** and ZIKV RNA levels were determined in tissue lysates with RT-PCR at two dpi **(D)**. N = 3–4 donors per condition, on average 12 explants per donor. Horizontal lines in the violin plots represent median and the 10^th^ and 90^th^ percentile cut-off. Statistical significance was determined using the Kruskal-Wallis test followed by Dunn’s post hoc test. * P<0.05, **P<0.01, ****P<0.0001.

Significantly higher ZIKV titers were found in supernatants of explants infected with ZIKV+DENV nAbs and ZIKV+hu4G2 compared to explants infected with only ZIKV or ZIKV+control at all the time points but mainly at two dpi (49.4-fold and 45.9-fold increase in median titer compared to only ZIKV, respectively, P < .0001, Fig 3A). Part of the explants were homogenized at six dpi for ZIKV RNA detection with RT-PCR and ZIKV RNA levels were significantly higher in explants infected with ZIKV+hu4G2 but not with ZIKV+DENV nAbs compared to only ZIKV at six dpi (12.2-fold and 2.9-fold increase in TCID_50_ equivalent, respectively, P = .03 and P = .99, Fig 3B).

We next studied the contribution of different FcγRs and FcRn on ADE of ZIKV in placental explants by pre-incubating the explants with monoclonal antibodies that block the interaction with IgG and FcγRs [37–41]. Furthermore, protein G was added to the ZIKV immune complexes prior to adding them to the explants to block the interaction between IgG and FcRn. We confirmed that adding protein G to ZIKV–DENV nAbs immune complexes did not inhibit ADE of ZIKV infection in the monocytic cell line U937 that is commonly used for *in vitro* ADE assays (S4 Fig). Since we mainly observed ADE of ZIKV infection in placental explants at two dpi, Fc-receptor blocking experiments were performed until two dpi.

Protein G significantly reduced ADE of ZIKV infection in placental explants (41-fold reduced median ZIKV titer and a 78-fold reduced median RNA levels, P < .0001, Fig 3C). Blocking FcγRII resulted in a 16-fold reduction in ZIKV titer in supernatants, but this difference was not statistically significant (P = .07).

Collectively, these data confirm that serum containing DENV antibodies can enhance ZIKV infection in placental explants and that this enhancement is FcRn dependent.

### ZIKV infects Hofbauer cells and trophoblasts in placental explants

**Fig 4:**
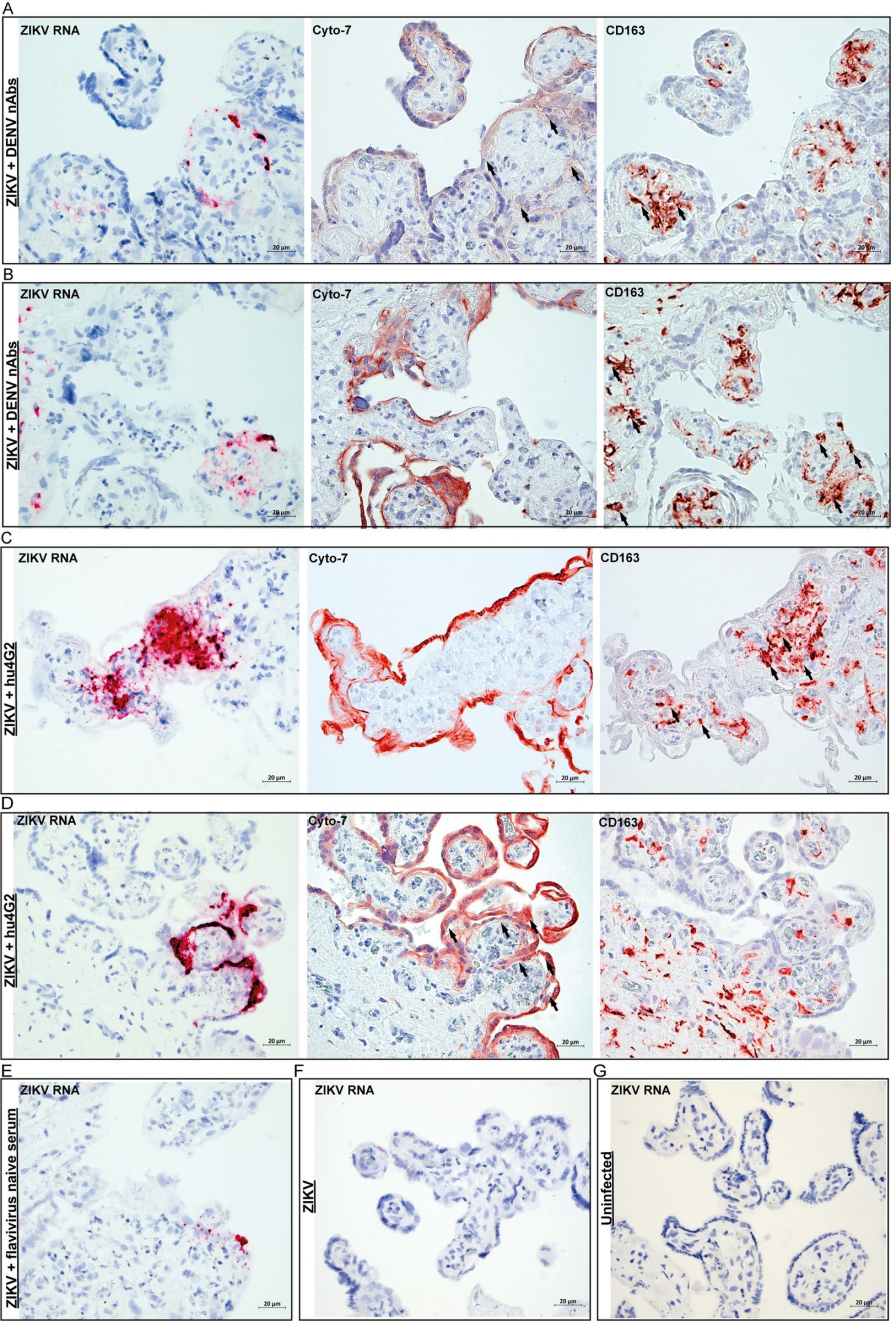
ZIKV infects Hofbauer cells and trophoblasts in placental explants in presence DENV nAbs. **A-D:** Representative pictures of ZIKV infection in Hofbauer cells (CD163) and trophoblasts (Cyto-7), in term human placental explants infected with ZIKV that was pre-incubated with human serum containing DENV-2 nAbs (**A&B)** or a humanized pan-flavivirus monoclonal antibody (hu4G2, **C&D)**. Arrows indicate cells stained with either Cyto-7 or CD163 that correspond with cells in which ZIKV RNA was detected. **E-G:** Staining for ZIKV RNA in placental explants infected with ZIKV+flavivirus naïve serum **(E)**, only ZIKV **(F)** or uninfected **(G)**. All tissues were stained six days after (mock) infection.

Next, we investigated which placental cells got infected with ZIKV in the placental explants. We performed ISH for ZIKV RNA in the placental explants that were infected with ZIKV, ZIKV+control, ZIKV+DENV nAbs and ZIKV+hu4G2 for six days. ZIKV RNA was more often detected and of higher intensity in placental explants infected with ZIKV+DENV nAbs or ZIKV+hu4G2 compared to explants infected with ZIKV or ZIKV+control in which ZIKV RNA signal was only sporadically detected (Fig 4A–4F). To determine which type of placental cells got infected with ZIKV, sequential slides were stained for CD163 and cytokeratin-7 as markers for HBCs and trophoblasts, respectively. In the explants infected with ZIKV+hu4G2 and ZIKV+DENV nAbs, ZIKV RNA was detected in HBCs (Fig 4A–4C) and in villous trophoblasts (Fig 4A). These findings demonstrate that during ADE of ZIKV infection in term placental explants, both villous trophoblasts and HBCs can get infected.

### Primary HBCs are permissive for ZIKV infection and ADE of ZIKV infection

To further confirm the role of HBCs and trophoblasts in ZIKV infection and ADE of ZIKV infection, we isolated and infected HBCs and trophoblasts from term human placentas.

**Fig 5:**
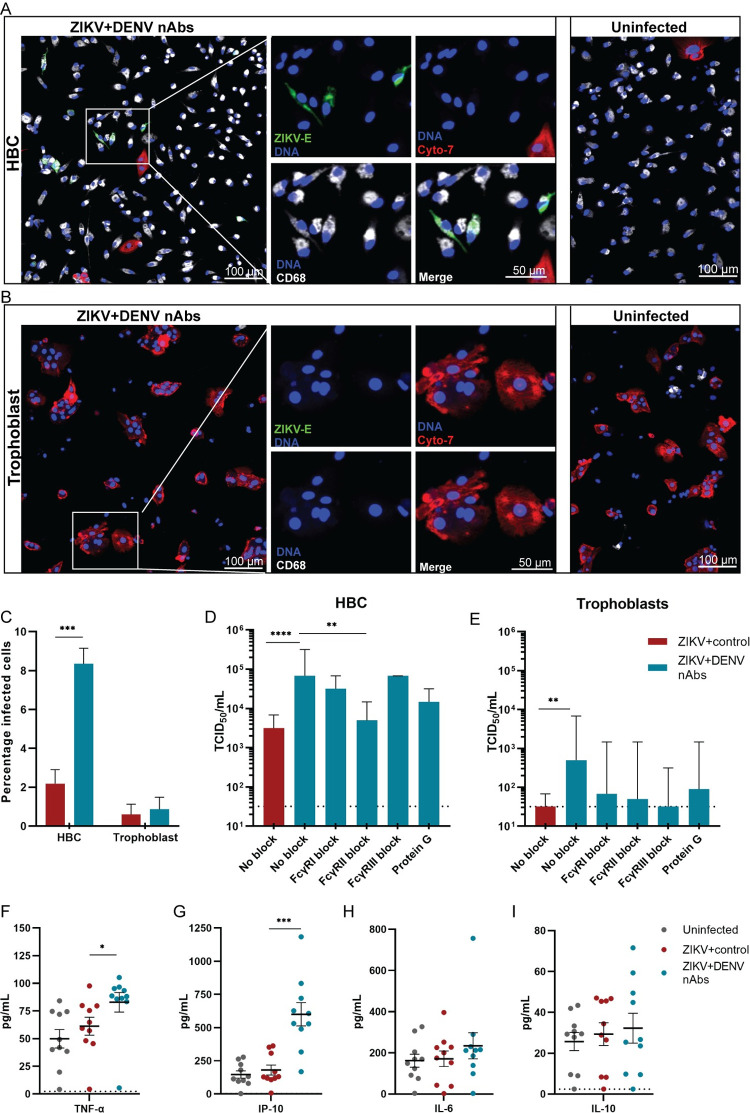
Primary Hofbauer cells are permissive for ZIKV infection and ADE of ZIKV infection. Hofbauer cells (HBCs) and trophoblasts were isolated from term human placentas and infected with ZIKV+flavivirus naive serum (ZIKV+control) or ZIKV+DENV nAbs at an MOI of 0.5 in presence or absence of FcγR blocking antibodies and protein G, for 48 hours. **A:** Confocal laser scanning microscopy image of ZIKV+DENV nAbs infected HBCs and uninfected HBCs. **B:** Confocal laser scanning microscopy image of ZIKV+DENV nAbs infected trophoblasts and uninfected trophoblasts. HBCs were visualized by fluorescent staining for CD68, trophoblasts for cytokeratin-7 (Cyto-7), ZIKV by staining for ZIKV envelope protein (ZIKV-E) and nuclei with Hoechst 33342 staining (DNA). **C:** Percentage of infection of HBCs and trophoblasts was determined with confocal laser scanning microscopy. Bars represent mean+SEM. Significance was determined with a Student’s T-test. **D&E:** ZIKV titers were determined in supernatants of HBCs and trophoblasts. Bars represent median+95%CI. Significance was determined using the Kruskal-Wallis test followed by Dunn’s post hoc test, comparing ZIKV+DENV nAbs without block to the other conditions. **F-I:** Cytokines were determined in the supernatants of HBCs with a multiplex bead-based assay. Each dot represents one value of experiments performed in triplicate/quadruplicate, lines represent mean ±SEM. Significance was determined using one-way ANOVA with Dunnett’s post hoc test. N = 2–3 donors per condition for all experiments. *P<0.05, ** P<0.01, ***P<0.001, ****P<0.0001.

Infection of HBCs with ZIKV+control resulted 2.2% infected cells while this increased to 8.8% when HBCs were infected with ZIKV+DENV nAbs (P = .0001, Fig 5A and 5C). Infection of HBCs with ZIKV+DENV nAbs also resulted in significantly enhanced ZIKV titers in supernatants compared to infection with ZIKV+control (21.5-fold increased median ZIKV titer at two dpi, P < .0001, Fig 5D). ADE of ZIKV infection in HBCs was partially inhibited by pre-treating the cells with FcγRII blocking antibodies (13.7-fold reduced median ZIKV titer in supernatant compared to no FcγRII-block, P = .003, Fig 5D). For trophoblasts, percentage of infected cells and viral titers in supernatants were negligibly low for cells infected with ZIKV+control (0.6% and median 32 TCID_50_/mL) and ZIKV+DENV nAbs (0.9% and median 498 TCID_50_/mL) (Fig 5B, 5C and 5E).

To determine the effect of ZIKV infection and ADE of ZIKV infection on cytokine production, we used a 13-plex bead-based assay to determine concentrations of cytokines and chemokines in the supernatants of HBCs and trophoblasts at two days post (mock) infection. ADE of ZIKV infection in HBCs significantly induced production of TNF-α and IP-10 compared to uninfected HBCs (1.7-fold increase and 4.1-fold-increase respectively, P = .014 and P = .0001, Fig 5F and 5G). No differences were seen for other cytokines, notably not for cytokines that are associated with intrinsic ADE such as IL-6 and IL-10 (Figs 5H, 5I and S5A) [42,43]. Cytokine concentrations in supernatants of trophoblasts that were infected with ZIKV+control or ZIKV+DENV nAbs did not differ from uninfected trophoblasts (S5B Fig).

## Discussion

**Fig 6:**
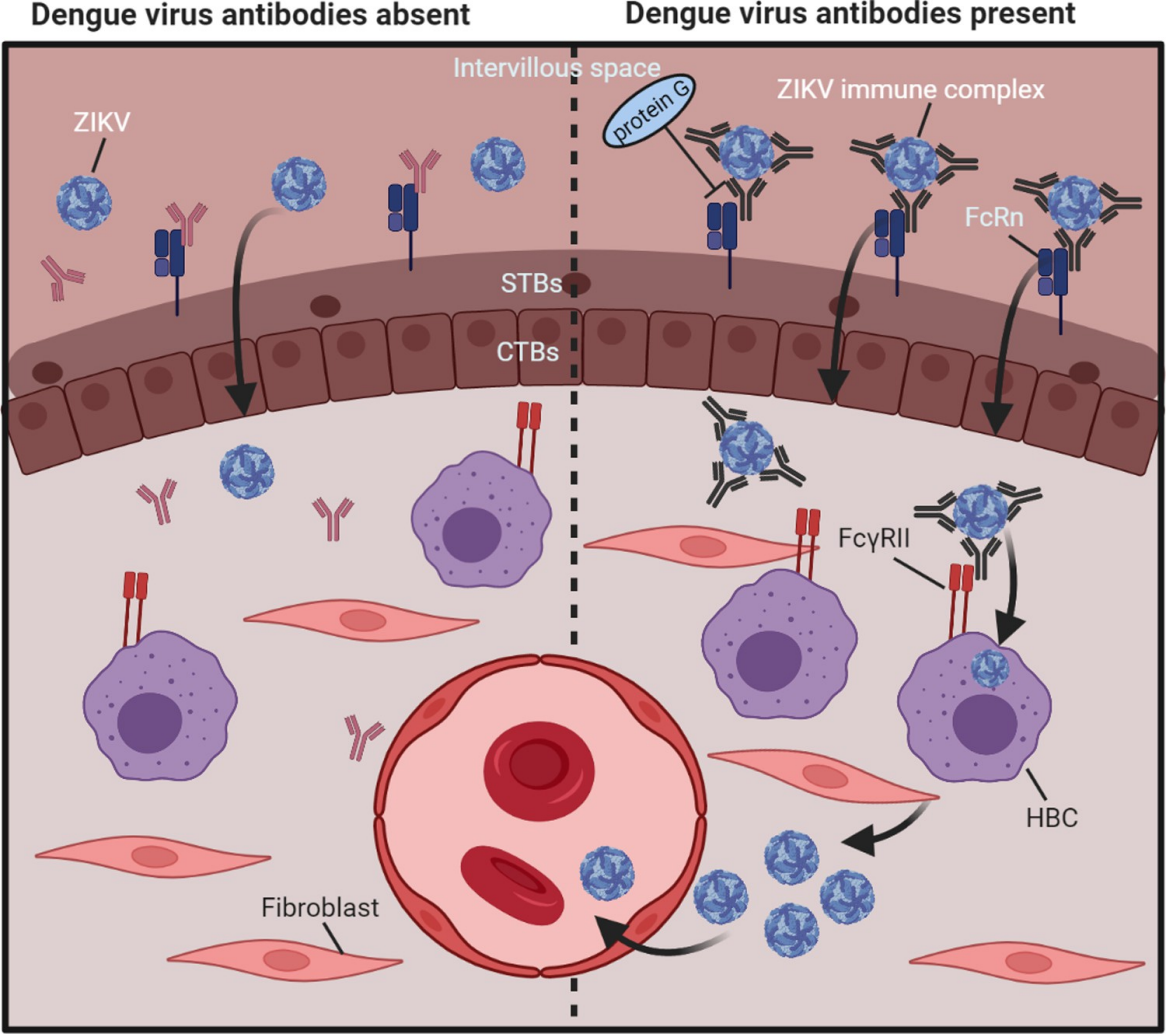
Proposed mechanism of ADE of ZIKV infection in the term human placenta. **Left panel:** In absence of cross-reactive DENV antibodies, ZIKV crosses the placental barrier less efficiently than in presence of cross-reactive flavivirus antibodies through a mechanism that is not fully elucidated yet. **Right panel:** In presence cross-reactive DENV antibodies, ZIKV immune complexes can be transported across the syncytiotrophoblasts layer through FcRn-mediated transcytosis. In the villus core, some non-neutralized complexes are taken up by the perivascular located HBCs through FcγRII, after which ZIKV can replicate in these cells. To reach the fetal circulation, ZIKV needs to subsequently cross the fetal endothelial barrier, possibly by infecting these cells. IVP; intervillous space, FcRn; neonatal Fc-receptor, STBs; syncytiotrophoblasts, CTBs; cytotrophoblasts, HBC; Hofbauer cell, FcγRII; Fcγ-receptor II. Created with Biorender.com.

Here, we demonstrate that *ex vivo* perfused human placentas efficiently take up ZIKV–DENV nAbs immune complexes and that this is likely FcRn-dependent. Furthermore, we show that ZIKV infection in placental explants and in HBCs is enhanced by anti-flavivirus antibodies. Collectively, these data support the hypothesis of enhanced ZIKV transplacental transmission through FcRn-mediated transcytosis of ZIKV–DENV nAbs immune complexes and through peripheral ADE of ZIKV infection in HBCs in the villus core as illustrated in Fig 6.

Because of the aforementioned limitations of placental explants and *in vivo* studies with pregnant mice, the pregnant NHP model is considered the most relevant to study transplacental pathogen transmission [5,44]. Compared to the pregnant NHP model, the *ex vivo* human placental perfusion model is a species specific, animal friendly and less expensive model that can be used to study transplacental pathogen transmission. This model has been previously used to study transplacental transmission of CMV, Coxsackie B-3 and ECHO-11 virus, however, transplacental transmission was not observed in these studies [45,46]. A limitation of this model is that the placenta can only be perfused for several hours. This limited perfusion time often does not allow for viral replication. Furthermore, perfusion experiments had to be performed with inactivated ZIKV because the placental perfusion model is situated in a biosafety level I laboratory. As expected with inactivated ZIKV and after a relative short perfusion time, we did not detect ZIKV RNA in the fetal circulation of perfused placentas. It is likely that viral replication in the villus core or endothelial cells is required for ZIKV to cross the endothelial barrier and reach the fetal circulation [47].

Besides ZIKV, FcRn-mediated transplacental transcytosis of virus-antibody immune complexes been observed for CMV [13]. Furthermore, studies have demonstrated that immune complexes in general can cross the placenta [48,49]. This indicates that FcRn-mediated placental uptake or transplacental transcytosis of pathogen-antibody immune complexes likely is a more generalized phenomenon that is not restricted to ZIKV. However, in most cases, the pathogen will be neutralized by the antibodies bound to it and will not be able to escape the syncytiotrophoblasts or will subsequently be phagocytosed and cleared by HBCs [13,50]. Because of antibody cross-reactivity, non-neutralized virus-antibody complexes can easily arise *in vivo* for flaviviruses such as ZIKV. The reason why ZIKV is the only flavivirus associated with birth defects might be that ZIKV is the only flavivirus that can infect fetal endothelial cells [51].

FcRn-mediated transplacental transcytosis can only occur from the second pregnancy trimester onwards, since IgG is not transported across the placenta before this time. The risk of ZIKV induced congenital malformations in humans, however, is highest when a ZIKV infection occurs during the first trimester of pregnancy when the placenta is still developing [52]. Therefore, FcRn-mediated transplacental transcytosis of ZIKV immune complexes might be an additional mechanism contributing to transplacental transmission of ZIKV in the second and third trimester of pregnancy, while in the first trimester, ZIKV can infect multiple proliferating placental cells [53]. The reason why increased fetal pathology has not been observed in DENV immune pregnant NHP that got infected with ZIKV could be that these NHP were infected in the first trimester of pregnancy when maternal IgG is not yet transported efficiently across the placenta [11,12,54].

Based on previous studies and clinical observations, HBCs seem to be the main target for ZIKV infection in the human placenta while cytotrophoblasts and especially syncytiotrophoblasts seem less permissive [2,55–59]. However, in first trimester mouse placentas and first- and second trimester human placental explants, ZIKV was mainly detected in trophoblasts [9,10,60]. Here, we found that during ADE of infection both trophoblasts and HBCs could be infected with ZIKV in term placental explants. After isolation of these cells from placental tissue, we found that HBCs are permissive for ZIKV infection and ADE of ZIKV infection while this was negligible for trophoblasts. Combining results from this study and previous studies, HBCs seem the main target for ZIKV infection and ADE of ZIKV. Trophoblasts mainly seem permissive for ZIKV infection early in pregnancy while later in pregnancy, they become more resistant to ZIKV infection [9,61–63].

It has been reported that type I and type III interferons are important in restricting ZIKV infection in HBCs and trophoblasts, respectively [58,64]. However, we did not observe evident induction of these interferons during ZIKV infection or ADE of ZIKV infection in either HBCs or trophoblasts. In general, we found that ZIKV infection and ADE of ZIKV infection results in modest induction or inhibition of the cytokines that we tested, possibly because of the relatively low percentage of HBCs and trophoblasts that got infected.

There are several studies that indicate that DENV pre-immunity might not have a negative impact on disease severity of a ZIKV infection and might even offer protection against ZIKV infection [65–67]. However, the data of this study indicates that caution is warranted for the potential harmful effects of cross-reactive DENV antibodies on transplacental transmission of ZIKV. This is relevant because of the co-circulation of ZIKV and DENV in many geographical regions and in the context of multivalent DENV vaccines that can induce an antibody repertoire that is partially cross-reactive with ZIKV [5,68]. Establishing the range of pre-existing DENV antibody titers that can increase the risk of transplacental ZIKV transmission *in vivo*, can be an important next step for risk assessment of vertical ZIKV transmission during pregnancy. Furthermore, we suggest that the *ex vivo* placental perfusion model is a highly relevant and animal friendly alternative for the pregnant NHP model to study transplacental pathogen transmission.

## Supporting information

S1 Fig β-propriolactone inactivated ZIKV can be detected with RT-PCR and *in situ* hybridization. **A**: β-propriolactone inactivated ZIKV (ZIKV-BPL) can still detected with RT-PCR for ZIKV RNA, albeit with a lower sensitivity (~3 CT-value’s lower). **B andC**; ZIKV **(B)** and ZIKV-BPL **(C)** can both be detected with *in situ* hybridization for ZIKV RNA after being incubated (MOI 2) with the monocytic cell line K562 for two hours on ice.
(TIF)

S2 Fig Selection of pooled sera for placental perfusion experiments. **A:** Thirty sera that did not contain ZIKV nAbs, from a ZIKV seroprevalence cohort, were tested for ADE potential by pre-incubation of the sera at a 1:100 dilution with ZIKV (MOI 0.5) prior to adding this to U937 cells for 48 hours. **B:** Sera marked with an asterisk in panel A were pooled and pre-incubated with ZIKV (MOI 0.5) at four different dilutions prior to adding them to U937 cells for 48 hours. Bars represent median ZIKV titers ±IQR in supernatants.
(TIF)

S3 Fig Uptake of ZIKV immune complexes by *ex vivo* perfused placentas is partially blocked by protein G. **A:** Protein G was added to ZIKV^BPL^+DENV nAbs in a concentration of 3 μg/mL and 9 μg/mL (N = 1 and N = 2 donors, respectively) and incubated for 60 minutes before adding this to the maternal circulation (MC) of the placental perfusion model. ZIKV RNA levels in the MC were determined every 15 minutes with RT-PCR up to 120 minutes. **B:** ZIKV RNA was detected in tissue biopsies taken from placentas that were perfused for 120 minutes. N = 2–3 donors per condition and 40–60 biopsies per condition. Horizontal lines represent median and the 10^th^ and 90^th^ percentile cut-off. Statistical significance was determined using the Mann-Whitney U test. **C:** ZIKV^BPL^+flavivirus negative serum (ZIKV^BPL^+control) and ZIKV^BPL^+DENV nAbs were circulated through the perfusion machine to which no placenta was attached to test for tube adherence of the immune complexes. ZIKV RNA levels in the MC were determined every 15 minutes with RT-PCR up to 90 minutes.
(TIF)

S4 Fig Adding protein G to ZIKV+DENV nAbs does not inhibit ADE of infection in U937 cells. U937 cells, expressing FcyR-I& -II, were infected with ZIKV (MOI 0.5) that was pre-incubated with flavivirus naïve serum (ZIKV+control) or serum containing DENV nAbs (both 1:250 dilution) with or without protein G. Cells were also pre-treated with FcγR blocking antibodies. ZIKV titers were determined in supernatants at two dpi. Bars represent median+95%CI. Significance was determined using the Kruskal-Wallis test followed by Dunn’s post hoc test, comparing ZIKV+DENV nAbs without block to the other conditions. * P < .05, ***P < .001.
(TIF)

S5 Fig No significant changes in cytokines produced by Hofbauer cells and trophoblasts during ZIKV infection. Cytokines were determined in the supernatants of Hofbauer cells (**A**) and trophoblasts (**B**), 48 hours after infection with ZIKV+control or ZIKV+DENV nAbs at an MOI of 0.5. Each dot represents one value of experiments performed in triplicate/quadruplicate, lines represent mean±SEM. Significance was determined using one-way ANOVA with Dunnett’s post hoc test. N = 3 donors per condition.
(TIF)

S1 Table Clinical characteristics of donors from whom placentas were used for perfusion experiments. (DOCX)

S2 Table Results from ZIKV and DENV-2 VNT assays and ZIKV and DENV NS1 IgG ELISA’s performed with sera used for enhancement experiments. (DOCX)
