# Supplementary figures and images for: Transplacental Zika virus transmission in ex vivo perfused human placentas
Source: PLoS Negl Trop Dis. 2022 Apr 20;16(4):e0010359. doi: 10.1371/journal.pntd.0010359 (PMC9060339; doi:10.1371/journal.pntd.0010359)

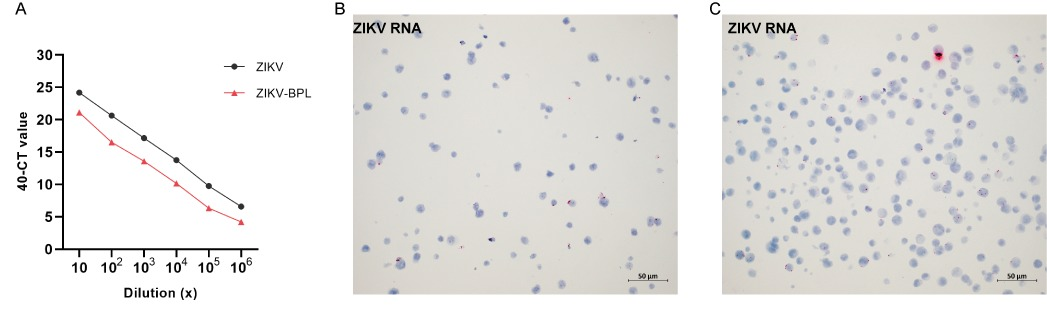

Supplement: S1 Fig — A: β-propriolactone inactivated ZIKV (ZIKV-BPL) can still detected with RT-PCR for ZIKV RNA, albeit with a lower sensitivity (~3 CT-value’s lower). B andC; ZIKV (B) and ZIKV-BPL (C) can both be detected with in situ hybridization for ZIKV RNA after being incubated (MOI 2) with the monocytic cell line K562 for two hours on ice. (TIF) [file pntd.0010359.s001.tif]

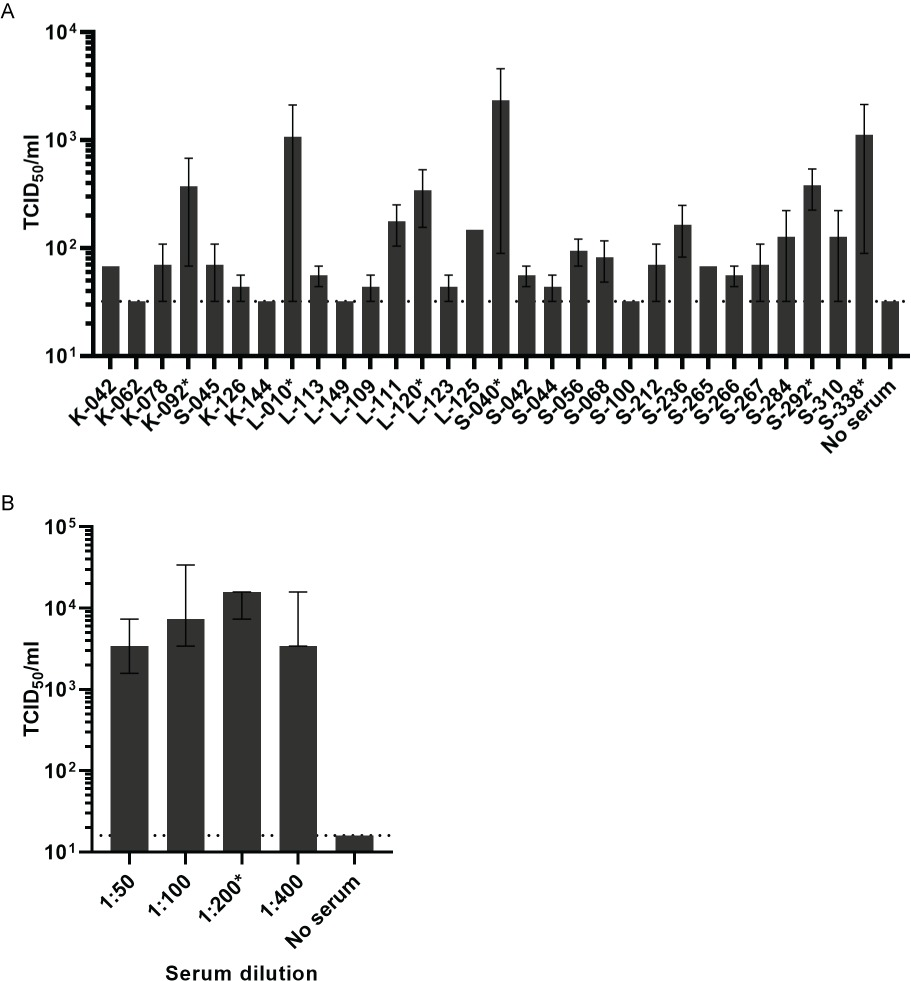

Supplement: S2 Fig — A: Thirty sera that did not contain ZIKV nAbs, from a ZIKV seroprevalence cohort, were tested for ADE potential by pre-incubation of the sera at a 1:100 dilution with ZIKV (MOI 0.5) prior to adding this to U937 cells for 48 hours. B: Sera marked with an asterisk in panel A were pooled and pre-incubated with ZIKV (MOI 0.5) at four different dilutions prior to adding them to U937 cells for 48 hours. Bars represent median ZIKV titers ±IQR in supernatants. (TIF) [file pntd.0010359.s002.tif]

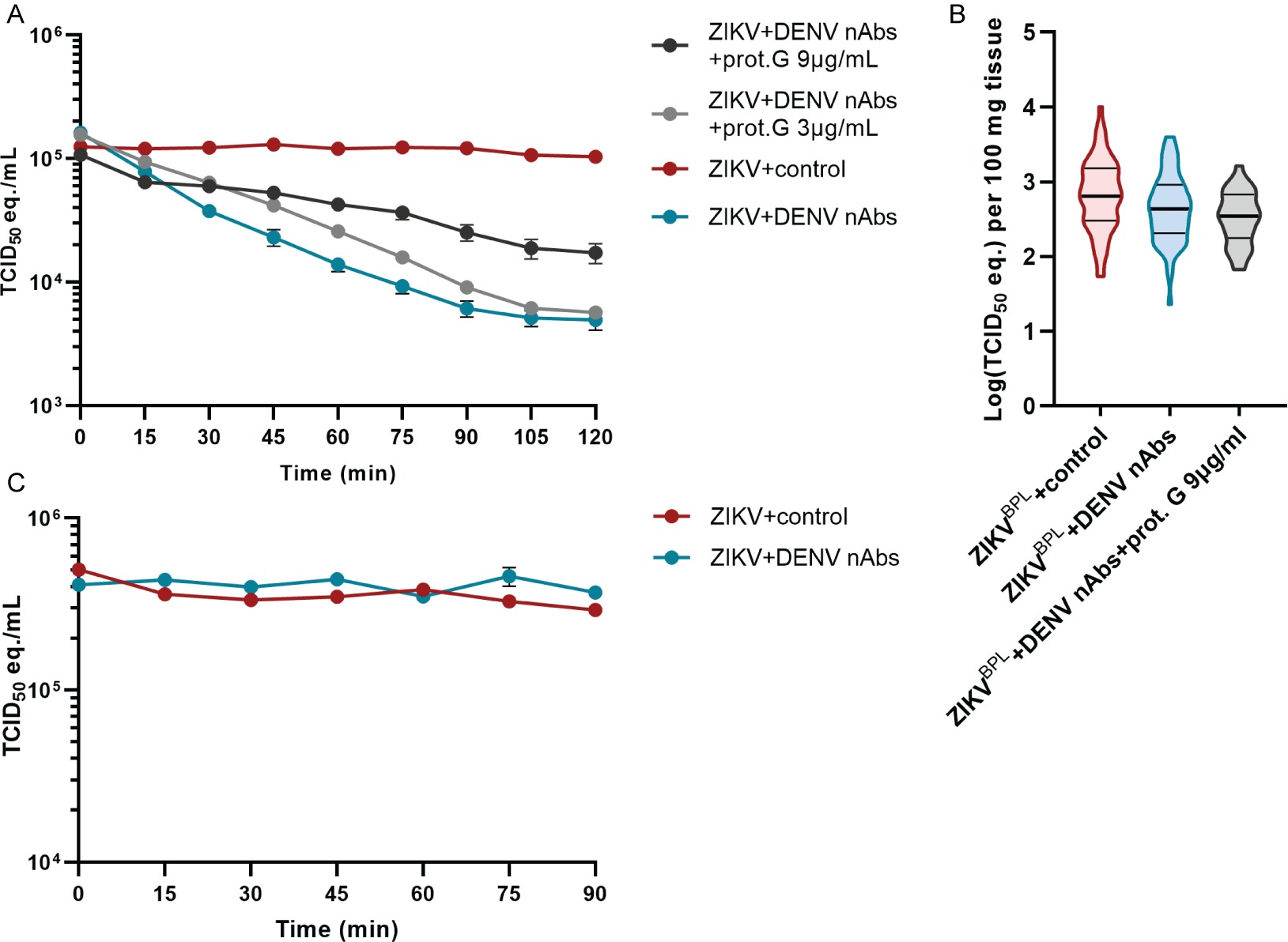

Supplement: S3 Fig — A: Protein G was added to ZIKVBPL+DENV nAbs in a concentration of 3 μg/mL and 9 μg/mL (N = 1 and N = 2 donors, respectively) and incubated for 60 minutes before adding this to the maternal circulation (MC) of the placental perfusion model. ZIKV RNA levels in the MC were determined every 15 minutes with RT-PCR up to 120 minutes. B: ZIKV RNA was detected in tissue biopsies taken from placentas that were perfused for 120 minutes. N = 2–3 donors per condition and 40–60 biopsies per condition. Horizontal lines represent median and the 10th and 90th percentile cut-off. Statistical significance was determined using the Mann-Whitney U test. C: ZIKVBPL+flavivirus negative serum (ZIKVBPL+control) and ZIKVBPL+DENV nAbs were circulated through the perfusion machine to which no placenta was attached to test for tube adherence of the immune complexes. ZIKV RNA levels in the MC were determined every 15 minutes with RT-PCR up to 90 minutes. (TIF) [file pntd.0010359.s003.tif]

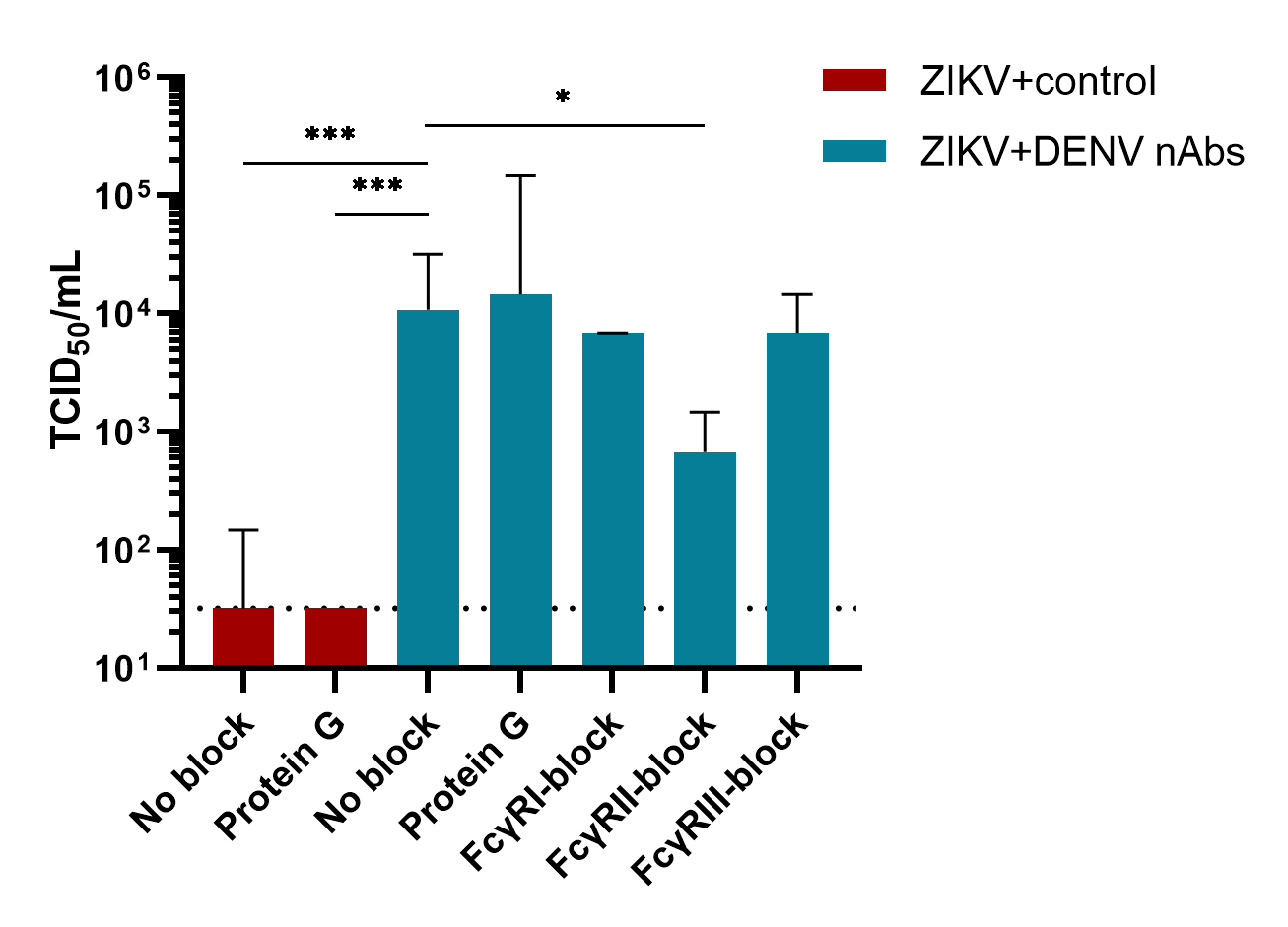

Supplement: S4 Fig — U937 cells, expressing FcyR-I& -II, were infected with ZIKV (MOI 0.5) that was pre-incubated with flavivirus naïve serum (ZIKV+control) or serum containing DENV nAbs (both 1:250 dilution) with or without protein G. Cells were also pre-treated with FcγR blocking antibodies. ZIKV titers were determined in supernatants at two dpi. Bars represent median+95%CI. Significance was determined using the Kruskal-Wallis test followed by Dunn’s post hoc test, comparing ZIKV+DENV nAbs without block to the other conditions. * P < .05, ***P < .001. (TIF) [file pntd.0010359.s004.tif]

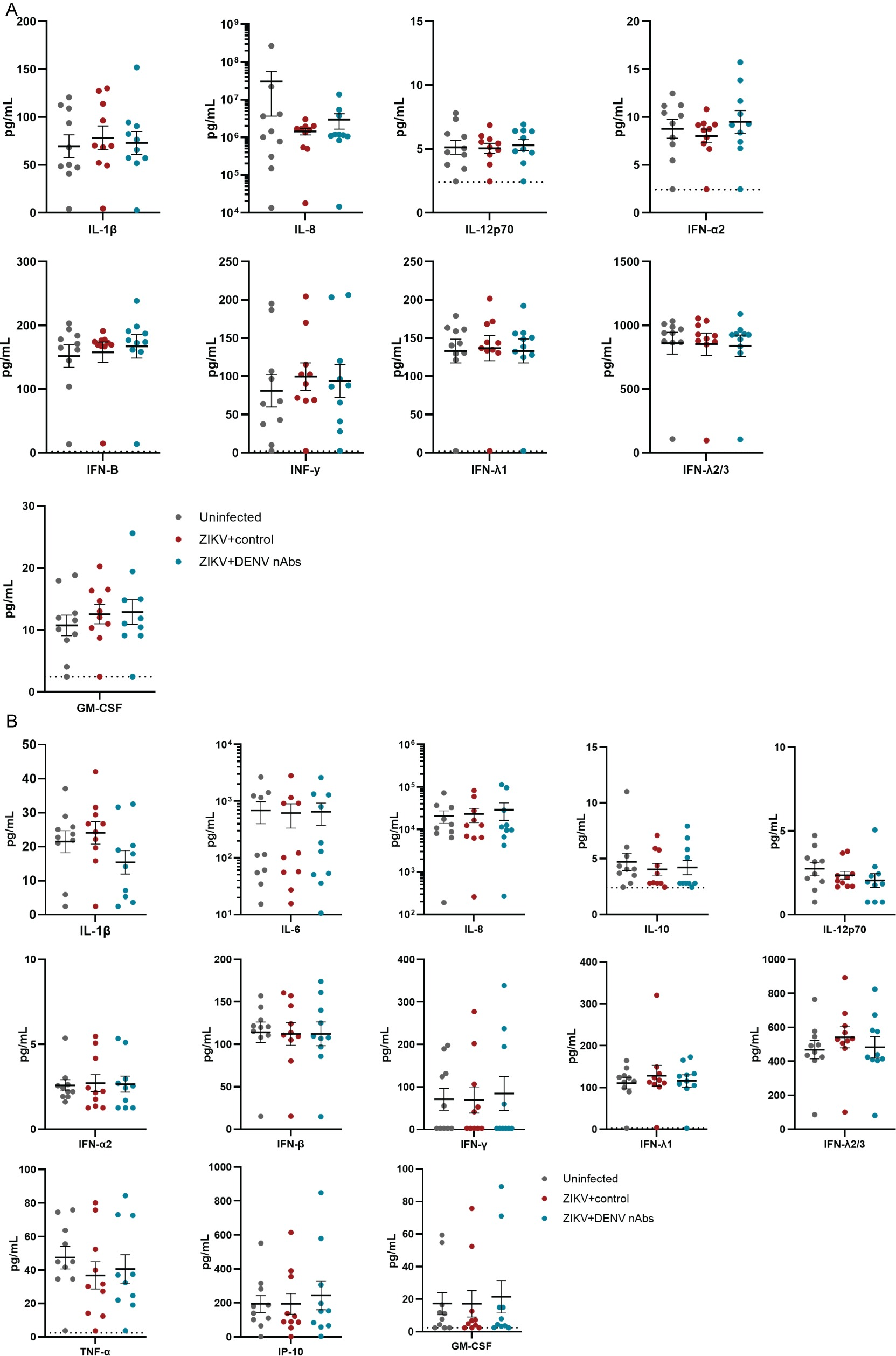

Supplement: S5 Fig — Cytokines were determined in the supernatants of Hofbauer cells (A) and trophoblasts (B), 48 hours after infection with ZIKV+control or ZIKV+DENV nAbs at an MOI of 0.5. Each dot represents one value of experiments performed in triplicate/quadruplicate, lines represent mean±SEM. Significance was determined using one-way ANOVA with Dunnett’s post hoc test. N = 3 donors per condition. (TIF) [file pntd.0010359.s005.tif]
