# Supplementary material for: Transplacental Zika virus transmission in ex vivo perfused human placentas
Source: PLoS Negl Trop Dis. 2022 Apr 20;16(4):e0010359. doi: 10.1371/journal.pntd.0010359 (PMC9060339; doi:10.1371/journal.pntd.0010359)
Supplement: S1 Table — (DOCX) [file pntd.0010359.s006.docx]

**Table S1:** Clinical characteristics of donors from whom placentas were used for perfusion experiments.

| **Donor** | **Condition** | **Maternal age, years** | **Gravidity** | **Parity** | **Gestational age, weeks+days** | **Fetal sex** | **Birth weight, g** | **Placental weight, g** |
| --- | --- | --- | --- | --- | --- | --- | --- | --- |
| 1 | ZIKV+control 120 min. | 34 | 2 | 1 | 39+1 | Male | 3090 | 650 |
| 2* | 1. ZIKV+control, 120 min.  2. ZIKV+DENV nAbs, 120 min. | 32 | 4 | 1 | 38+4 | Female | 3540 | 737 |
| 3 | ZIKV+DENV nAbs, 120 min. | 38 | 6 | 3 | 39+0 | Female | 3685 | 617 |
| 4 | ZIKV+DENV nAbs, 120 min | 30 | 2 | 1 | 39+2 | Male | 3320 | 551 |
| 5* | 1. ZIKV+DENV nAbs, 120 min.  2. ZIKV+DENV nAbs+Prot. G 3µg/mL, 120 min. | 38 | 5 | 3 | 38+4 | Male | 3625 | 726 |
| 6* | 1. ZIKV+control, , 120 min.  2. ZIKV+DENV nAbs+Prot. G 9µg/mL, 120 min. | 34 | 2 | 1 | 39+3 | Male | 4005 | 657 |
| 7 | ZIKV+DENV nAbs+Prot. G 9µg/mL, 120 min. | 27 | 5 | 1 | 38+4 | Female | 3340 | 691 |
| 8 | ZIKV+DENV nAbs, 40 min. | 28 | 2 | 1 | 39+0 | Male | 3545 | 736 |
| 9* | 1. ZIKV+control, 40 min.  2. ZIKV+DENV nAbs, 40 min. | 33 | 9 | 5 | 38+6 | Male | 4040 | 762 |
| 10 | ZIKV+control, 40 min. | 35 | 3 | 2 | 39+3 | Female | 3385 | 667 |

In placentas from donors marked with *, two cotyledons of the same placenta could be perfused at the same time and therefore, two conditions could be tested with the same placenta.
