# Supplementary material for: Transplacental Zika virus transmission in ex vivo perfused human placentas
Source: PLoS Negl Trop Dis. 2022 Apr 20;16(4):e0010359. doi: 10.1371/journal.pntd.0010359 (PMC9060339; doi:10.1371/journal.pntd.0010359)
Supplement: S2 Table — (DOCX) [file pntd.0010359.s007.docx]

**Table S2:** Results from ZIKV and DENV-2 VNT assays and ZIKV and DENV NS1 IgG ELISA’s performed with sera used for enhancement experiments.

| **Serum** | **ZIKV nAb titer** | **ZIKV NS1 IgG ELISA ratio** | **DENV-2 nAb titer** |
| --- | --- | --- | --- |
| L-010 | <8 | 4.14 | 161 |
| L-055 | <8 | 3.99 | 256 |
| L-120 | <8 | 2.31 | 128 |
| S-040 | <8 | 1.12 | 161 |
| S-292 | <8 | 4.05 | 128 |
| S-338 | <8 | 4.63 | >1024 |

Cut-off for positive ZIKV and DENV-2 VNT result: titer >1:32
Cut-off for positive ZIKV and DENV NS1 IgG ELISA result: ratio >1.1
